# In Vitro-Selected Clones of the Halophyte *Arthrocaulon macrostachyum* Display Enhanced Salinity Stress Tolerance

**DOI:** 10.3390/plants14081164

**Published:** 2025-04-09

**Authors:** Ghofrane Atrous, Abel Piqueras, Pedro Diaz-Vivancos, Ana Hernández-Cánovas, Karim Ben Hamed, José A. Hernández, Gregorio Barba-Espín

**Affiliations:** 1 Group of Fruit Trees Biotechnology, Department of Plant Breeding, Centro de Edafología y Biología Aplicada del Segura (CEBAS-CSIC), Campus Universitario de Espinardo, 30100 Murcia, Spain; ghofrane.atrous@um.es (G.A.); piqueras@cebas.csic.es (A.P.); pdv@cebas.csic.es (P.D.-V.); ahcanovas@cebas.csic.es (A.H.-C.); 2 Centre of Biotechnology of Borj Cedria, Laboratory of Extremophile Plants, BP 95, Hammam-Lif 2050, Tunisia; karim.benhamed@cbbc.rnrt.tn; 3 Faculty of Mathematical, Physical and Natural Sciences of Tunis, University of Tunis El Manar, Campus Universitaire El-Manar, Tunis 2092, Tunisia; 4 Manouba School of Engineering, University of Manouba, Manouba 2010, Tunisia

**Keywords:** antioxidant metabolism, *Arthrocaulon macrostachyum*, clonal halophyte, oxidative stress, salinity tolerance

## Abstract

Halophytes hold significance for soil desalination and co-cultivation in farming systems. A major impediment to their use is the standardization of their performance, since halophytes are mainly wild plants, in addition to the need for a constant supply of the most suitable species. In this work, using highly salt-tolerant clones of *Arthrocaulon macrostachyum* obtained previously from in vitro micropropagation and selection, we compared the physiological and biochemical responses of these clones and their wild counterparts to high salinity levels (428 mM NaCl) under glasshouse conditions. In vitro-derived clones displayed a superior biomass production (27%) and higher chloride concentration in the shoot (28%), compared to the wild plants. On the other hand, wild specimens showed more stress symptoms and a less efficient photosynthesis, which was correlated with higher levels of oxidative stress and with a remarkable induction of peroxidase activity. Therefore, a higher incidence of salinity-related oxidative stress in the wild halophytes in comparison to the clones is concluded. This represents the first ex vitro evaluation of halophyte clones selected by means of micropropagation and provides insights into the salinity tolerance mechanisms of *A. macrostachyum*.

## 1. Introduction

Halophytes are plant species that thrive in naturally saline environments, capable of completing their life cycle in soil salinities of at least 200 mM NaCl [1,2]. While they constitute approximately 2% of all terrestrial plant species, they are found across half of the higher plant families and exhibit a significant diversity in their morphological forms [2]. Nowadays, halophytes appear as a profitable alternative to traditional crops, whereas there is also increasing evidence showing their capability to restore degraded soils [3,4]. Therefore, they are considered a valuable tool to ensure food security and diversification, having a key role within the context of sustainability and climate change; particularly, halophytes are useful under soil and water salinization and freshwater scarcity conditions in agricultural systems [3,4,5,6,7].

One significant consequence of salt stress is the occurrence of oxidative damage, mediated by the excessive production of reactive oxygen species (ROS), including hydroxyl radicals, superoxide, and hydrogen peroxide [8,9]. Numerous morphological, physiological, biochemical, and molecular adaptations have been identified in halophytic plants that enable them to cope with saline conditions [1]. These adaptive strategies include the preservation of the photosynthetic apparatus through chlorophyll production, and the modulation of carotenoid levels and ROS levels. Additionally, the activation of enzymatic antioxidants, such as superoxide dismutase, peroxidases, and catalases, and the accumulation of non-enzymatic antioxidants and compatible solutes, may play a key role [1,2].

Recent molecular research has led to the classification of the halophyte *Arthrocnemum macrostachyum* into two distinct genera: *Arthroceras subterminale* and *Arthrocaulon macrostachyum* [10,11]. The latter species is indigenous to the salt marshes of the Mediterranean region [12,13,14]. Within the Iberian Peninsula, *A. macrostachyum* is found in both inland and coastal areas in the southern regions [15]. This halophyte is a succulent plant characterized by jointed stems with fleshy segments and reduced, fused leaves [15]. In terms of salt tolerance, it exhibits a broad range of soil salinity tolerance, accommodating concentrations from 170 to 510 mM NaCl [16]. Due to its significant capacity for sodium accumulation, *A. macrostachyum* has been identified as a potential candidate for the phytoremediation of moderate and high saline and sodic soils [17,18].

There is a growing body of research dealing with the plant tissue culture of halophytes, with aims ranging from the propagation of endangered species to the stimulated production of bioactive compounds or the multiplication of species with limited sexual and vegetative propagation [19,20]. Shoot multiplication techniques are widely utilized for plant micropropagation. In halophytes, shoot multiplication has been used for generating clones from selected specimens based on their salinity tolerance [21,22,23,24,25,26]. Variation, similar to that observed in natural ecosystems, can be obtained through tissue culture by means of somaclonal variation [27]. Somaclonal variation has been applied as a tool in breeding programs of major cash crops, generating genetic diversity and supporting the development of new genotypes resistant to different stresses [28]. However, there is a lack of studies that link micropropagation of halophyte clones with ex vitro evaluation of the generated specimens prior to ulterior uses in breeding and farming systems.

In a previous work, we obtained highly salt-tolerant clones of *A. macrostachyum* from in vitro micropropagation and selection [29]. In the present study, we conducted a physiological and biochemical evaluation of these clones in comparison with their wild counterparts, in response to salinity and under glasshouse conditions. The clone population used was expected to confer a higher phenotype homogeneity and salinity tolerance under ex vitro cultivation. This represents the first ex vitro evaluation of halophyte clones selected by means of micropropagation and provides insights into the salinity tolerance mechanisms of *A. macrostachyum*.

## 2. Methods

### 2.1. Plant Material and Glasshouse Cultivation

Plants of *A. macrostachyum* L. of two different origins were used. On one hand, clones derived from in vitro multiplication in a previous study [29] were grown for four months after their acclimatization to ex vitro conditions. On the other hand, four-months-old plants (from seed germination) were purchased in a plant nursery (“Viveros Muzalé”, Abanilla, Murcia, Spain). Both groups of plants (from now onwards IPs for in vitro-derived plants; and NPs for nursery plants) were transplanted into pots (15 cm diameter × 20 cm height) and filled with 3.4 L of a mix of garden soil and perlite particle size 1–5 mm) (1/5, *v*/*v*).

The pots were placed at the glasshouse facilities of the University of Murcia (Espinardo, Region of Murcia, Spain). Initially, plants were pruned lightly to a similar shape and size and kept for two weeks to adapt them to the new environmental conditions. Subsequently, the experiment was carried out from March to June 2023 over 10 weeks. Two independent repetitions were conducted with a one-week interval. Relative humidity was kept at 60%, whereas temperature oscillated between 18 (night) and 34 °C (day) with a mean daily temperature of 25 °C; this temperature range was selected to resemble oscillation found under natural conditions.

A drip irrigation system using one 4 L/h dripper per pot was installed to supply water (100 mL per day and pot). In addition, plants were fertilized with 500 mL of 1/2 Hoagland solution containing 428 mM NaCl, starting at day 0 and then every two weeks, until week 10. On the day of fertilization and the previous and subsequent days, water irrigation was interrupted.

The different analyses were performed on samples taken at two-week intervals (1 to 10 weeks), except for the mineral nutrient determination, which was performed uniquely at the end of the experiment (week 10).

### 2.2. Determination of Biomass Production and Electrical Conductivity (EC)

The fresh weight (FW) of the plant aerial part was measured at the end of the experiment, whereas shoot dry matter (DM) was calculated at different times in branch segments as the difference between fresh and dry weights after 48 h incubation at 60 °C.

Over the course of the experiment, the EC of the leachate, the irrigation water, and the nutrient solution containing NaCl were measured using a conductivity meter and results were expressed as mS cm^−1^.

### 2.3. Determination of Chlorophyll Fluorescence

Chlorophyll fluorescence was measured in plant branches using a chlorophyll fluorimeter (IMAGIM-PAM M-series, Heinz Walz, Effeltrich, Germany). Firstly, plants were incubated in the dark for 20 min, then selected branches were cut and introduced in the fluorimeter for the determination of the minimum and maximal fluorescence yields. Kinetic analyses were conducted with actinic light (81 µmol quanta m^−2^ s^−1^ PAR) and repeated pulses of saturating light at 2700 µmol quanta m^−2^ s^−1^ PAR for 0.8 s, at intervals of 20 s. The following variables were determined: effective PSII quantum yield [Y(II))]; non-photochemical quenching (NPQ); and coefficients of photochemical quenching (qP) and non-photochemical quenching (qN) [30].

### 2.4. Antioxidant Enzyme Activities

Enzyme extraction was performed in plant material previously stored at −80 °C. In brief, apical branch samples (1 g) were ground into a powder in the presence of liquid nitrogen, and then extracted in a medium (1/5, *w*/*v*) containing 50 mM Tris-acetate buffer (pH 6.0), 2 mm cysteine, 0.1 mM EDTA, and 0.2% (*v*/*v*) Triton X-100. Subsequently, extracts were centrifuged at 10,000× *g* for 20 min at 4 °C, and the generated supernatant filtered through Sephadex NAP-10 columns (GE Healthcare, Chicago, IL, USA). The activities were assayed using a UV/Vis V-630 Bio spectrophotometer (Jasco, Tokyo, Japan). Superoxide dismutase activity (SOD; EC 1.15.1.1) was assayed by the ferricytochrome c method using xanthine/xanthine oxidase as the source of superoxide radicals [31]; catalase activity (CAT; EC 1.11.1.6) was assayed according to [32]; peroxidase activity (POX; EC. 1.11.1.7) was assayed according to [33]. Protein concentration was determined according to [34] using a plate reader (Epoch2, BioTek, Winooski, VT, USA) and bovine serum albumin as standard.

### 2.5. Antioxidant Activity Assay

Antioxidant activity was measured using 1,1-diphenyl-2-picrylhydrazyl (DPPH) inhibitory activity [35] with minor modifications, in plant material previously lyophilized. In brief, a DPPH solution was prepared in methanol at a concentration of 0.6 mM and an absorbance of 0.68 ± 0.05 at 517 nm. The mixture was mixed vigorously and incubated at room temperature in the dark for 30 min. Subsequently, the absorbance of the samples was measured at 517 nm using a UV/Vis V-630 spectrophotometer (Jasco). The percentage of free radical inhibition activity of the extracts was calculated. Butylated hydroxytoluene was used as a standard reference for comparison, and the antioxidant activity was expressed using an IC50 (µg/mL) value, which corresponds to the concentration of the compound required to scavenge 50% DPPH free radical.

### 2.6. Lipid Peroxidation

The extent of lipid peroxidation was calculated as the concentration of thiobarbituric acid-reactive substances (TBARS), as reported [36,37], in plant material previously stored at −80 °C. The concentration of TBARS was determined from the extinction coefficient 155 mM^−1^ cm^−1^.

### 2.7. Mineral Nutrient Analysis

The plant material (whole aerial part) and soil samples were collected at the conclusion of the experiment. Samples were processed as described [7] and submitted to the Ionomic Services of CEBAS-CSIC for the analysis of macro- and micronutrient levels [Inductively Coupled Plasma–Optical Emission Spectrometry (ICP–OES) using a ICAP 6000SERIES spectrometer (Thermo Scientific, Madrid, Spain)] and of anions [ion-chromatography (Metrohm Ltd., Herisau, Switzerland)], according to standardized protocols.

### 2.8. Statistical Analysis

All analyses were performed with at least four biological replicates, each replicate consisting of samples from an individual plant. The data were analyzed by one-way ANOVA using the SPSS 20.0 software (SPSS Inc., 2002, Chicago, IL, USA), and presented as the mean ± the standard error.

## 3. Results and Discussion

In the present work, we aimed at assessing whether in vitro selection of clones of the halophyte *A. macrostachyum* may improve physiological and/or biochemical responses of the plant under saline conditions. To test this hypothesis, we measured different growth parameters (biomass production, photosynthesis), minerals, and antioxidant activities, both in IPs (in vitro-derived plants) and NPs (nursery plants), under saline conditions.

### 3.1. Growth-Related Measurements

**Figure 1:**
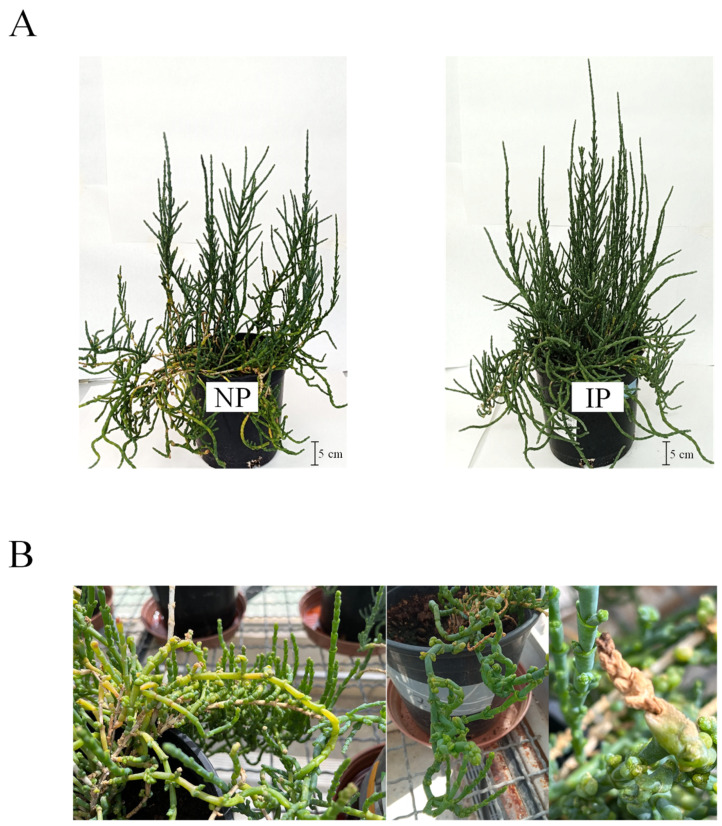
Nursery (NP) and in vitro-derived (IP) plants after 10 weeks of the experiment. (**A**) Overall appearance of plants. (**B**) Close-up view of NP showing yellow and brown decoloration and shape abnormalities.

At the conclusion of the experimental period, visual observations revealed that IPs exhibited more robust growth compared to NPs, with a greater number of branches and increased height (Figure 1A). In terms of biomass, IPs demonstrated a 27% higher aboveground biomass than NPs (469 g vs. 375 g). Furthermore, NPs exhibited morphological abnormalities and symptoms indicative of salt stress, which were absent in IPs (Figure 1B). These findings suggest that IPs exhibited greater tolerance to the experimental conditions, as evidenced by enhanced biomass accumulation and the absence of salt-induced symptoms, in contrast to NPs.

**Figure 2:**
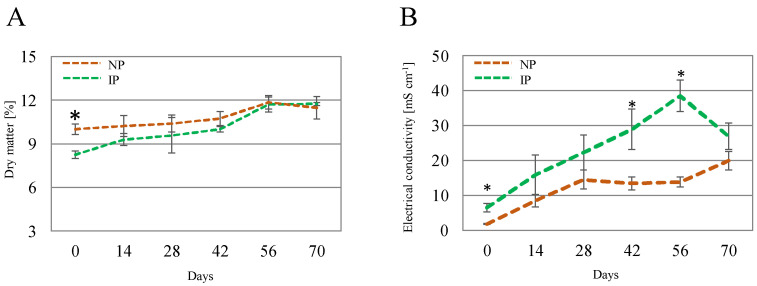
Shoot dry matter (**A**) and electrical conductivity of the leachate (**B**) for nursery (NP) and in vitro-derived (IP) plants at different times. Data are shown as the mean ± the standard error (n = 4). Asterix symbols (*) represent significant differences for pairwise comparisons according to one-way ANOVA.

Concerning shoot DM, it displayed variations over time and between IPs and NPs (Figure 2A); at time 0, DM for IPs had a value of 8.24%, whereas values for NPs were significantly higher (10.0%). This can be linked to a hyperhydricity phenomenon derived from the in vitro origin of the IP [38]. This difference gradually narrowed, with similar values at the end of the experiment for IPs (11.7%) and NPs (11.5%). The electrical conductivity (EC) of the leachate (Figure 2B) exhibited elevated levels, which can be attributed to the high salinity of the salinized nutrient solution employed, with a conductivity of 41.2 mS cm^−1^. Superior EC values in IPs than in NPs were found at some points, which suggests a more efficient uptake of nutrients for NPs; nevertheless, there was a drop in EC values for IPs at the last time point.

### 3.2. Mineral Nutrients

Table 1 and Table 2 displays the values in the soil and in the plant for Na, Cl, and for the rest of mineral nutrients and anions for which significant differences were observed. Soil Na and Cl remained invariable (Table 1). In contrast, IP shoots displayed a 28% increase in Cl when compared to NP samples (Table 2). This phenomenon may be attributed to the enhanced growth of IPs, as halophytic species exhibit a positive correlation between shoot chloride (Cl^−^) concentration and plant biomass [39]. This relationship is associated with the plant’s requirements for osmotic adjustment and optimal growth. Furthermore, Na concentration in the shoots remained consistent between both plant groups. (Table 2). *A. macrostachyum* utilizes Na to maintain cell turgor, promoting photosynthetic competence and plant growth [16,17]. Thus, these results suggest that differences in growth between NPs and IPs may be linked to other factors rather than the modulation of Na homeostasis. Nonetheless, taking into consideration the higher biomass accumulation of IPs mentioned above (27%), a superior desalting capacity can be deducted for these plants, which would account for 27% in the case of Na, and 44% for Cl. Moreover, in the last few years, Cl has been pointed out as beneficial when accumulated to macronutrient levels in plant tissues [40], revealing novel biological functions that improve plant growth, plant water relations, photosynthesis, and water-use efficiency [41,42]. In terms of applicability, these results highlight the potential of in vitro propagation for providing superior clones for desalinization purposes.

On the other hand, concentrations of nitrate, phosphate, and sulfate ions were significantly higher in NPs (Table 2), whereas these nutrients were unchanged in the soil. Moreover, Ca, Fe, and some micronutrients increased in soil associated with NPs (Table 1). In this regard, salinity may modulate nutrient uptake efficiency through both osmotic stress and ion competition mechanisms [43]. Moreover, the solubility of macro- and micronutrients in soil is affected by factors such as oxidation-reduction reactions and pH [44]. Thus, further experimentation will be needed to address the complex relationship between the halophyte, the soil, and plant nutrition.

### 3.3. Chlorophyl Fluorescence Parameters

**Figure 3:**
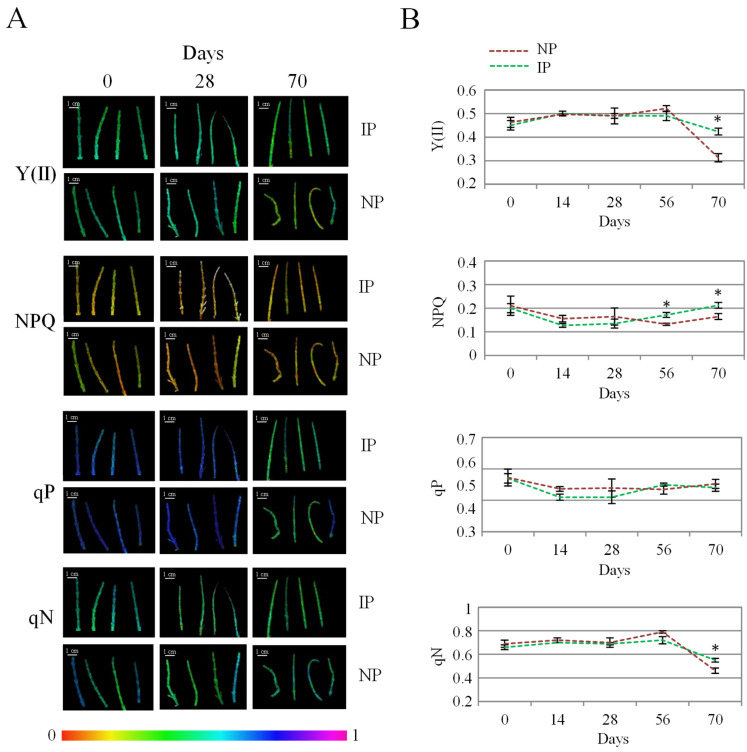
Evolution of photochemical [Y(II) and qP] and non-photochemical quenching (NPQ and qN) values for nursery (NP) and in vitro-derived (IP) plants at different times. (**A**) Colored images of apical shoot segments representing fluorescence intensity from black (0) to magenta (1) for the different variables at selected time points. (**B**) Data recorded at the different times, representing the mean  ±  the standard error (n  =  4). Asterix symbols (*) represent significant differences for pairwise comparisons according to one-way ANOVA.

Chlorophyll fluorescence analysis is a widely employed technique to evaluate the effect of stress on photosynthesis. In this work, the evolution of the chlorophyl fluorescence data was recorded to compare the state of photosynthesis in both plant groups (Figure 3). Overall, no major changes occurred over time, which indicates that salinity did not alter photosynthesis markedly, as reported in this species at different salinity levels [16]. By the end of the experiment, IP displayed significantly higher values for Y(II) and for the non-photochemical quenching variables, NPQ and qN (Figure 3), indicating a more efficient photosynthesis at this stage for IPs. In this regard, Y(II) indicates the proportion of the light absorbed by chlorophyll associated with PSII that is used for photochemistry [45]. On the other hand, non-photochemical variables correspond to the excess energy dissipated as heat by regulated mechanisms [46]; therefore, higher levels of non-photochemical quenching parameters can be associated with a better functioning of photosynthesis-related defense mechanisms in the halophyte under a stressful situation [7]. It is reported that halophytes hold the remarkable ability to protect net photosynthesis and stabilize both PS I and PS II under saline conditions, this way avoiding overproduction of ROS in the chloroplast associated with oxidative stress [47,48]. In addition, chloride accumulation has been related to a more efficient photosynthesis in halophytes by maintaining osmotic balance, stabilizing enzyme activity, and regulating stomatal function and photochemical reactions [49]. Thus, the fact that IPs displayed a more efficient photosynthesis than NPs under our experimental conditions is a significant feature that can result from the clonal origin of IP specimens and that can be related to their higher Cl accumulation in the shoot, which in turn could redound on the higher biomass observed for IPs.

### 3.4. Antioxidant Metabolism and Oxidative Stress

**Figure 4:**
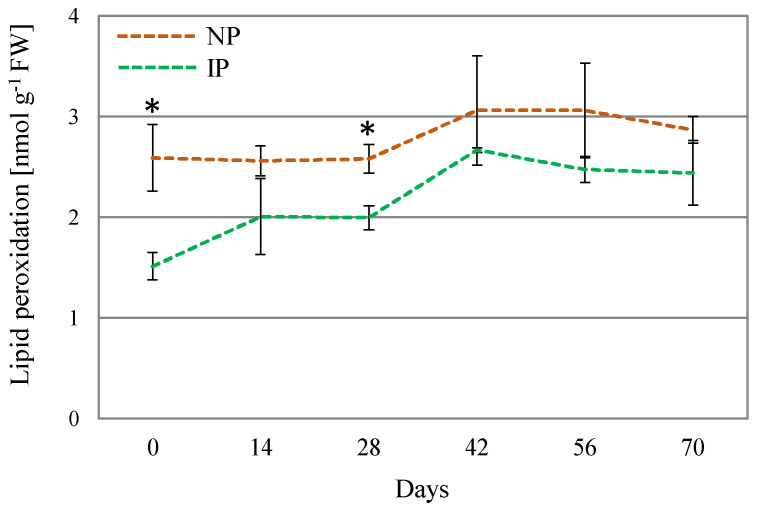
Lipid peroxidation levels for nursery (NP) and in vitro-derived (IP) plants at different times. Data are shown as the mean ± the standard error (n = 4). Asterix symbols (*) represent significant difference for pairwise comparison according to one-way ANOVA.

Oxidative stress is often linked to salt and water stress, via over-accumulation of ROS, which can oxidize amino acid residues in proteins, unsaturated fatty acids in cell membranes, and DNA molecules, thus, causing cellular damage [50]. The determination of lipid peroxidation is regarded as a suitable oxidative stress marker [51]. In this work, lipid peroxidation levels were found to moderately increase in NPs with respect to IPs (Figure 4), which denotes a higher level of oxidative stress damage on these plants. Nevertheless, the detected levels can be considered low for this species, since approximately 100-fold [52] and 10-fold higher levels [53] have been reported in *A. macrostachyum* plants collected in natural habitats. It is hypothesized that these significant differences arise from the plants’ origin, as those growing in natural environments are subjected to more adverse environmental conditions compared to those cultivated under glasshouse conditions. Moreover, no differences in DPPH activity were found between IPs and NPs. The DPPH method is used for the estimation of the non-enzymatic antioxidants in the plant extract; therefore, differences in growth and physiology between IPs and NPs cannot be attributed to modulation of non-enzymatic antioxidants. In this sense, in *Crithmum maritimum* plants, salinity did not affect the content of non-enzymatic antioxidants such as phenolic compounds [54]. On the other hand, in *A. macrostachyum* plants growing in mixed cultivation with tomato plants under greenhouse conditions, an accumulation of caffeoyl tartaric acid was observed [6]. These contrasting results are in agreement with our observation that the content in non-enzymatic antioxidants may be dependent on both plant species and growing conditions.

**Figure 5:**
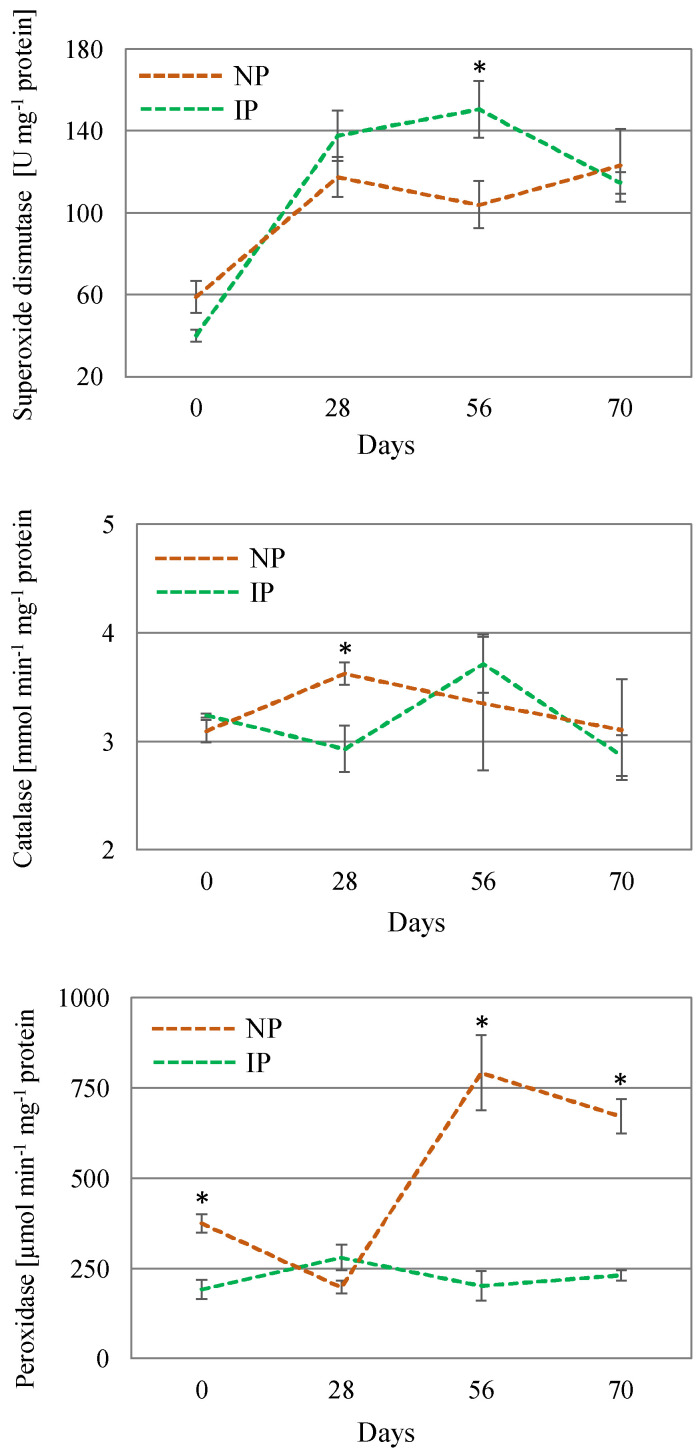
Activity of the enzymes catalase, peroxidase, and superoxide dismutase in shoot extracts of nursery (NP) and in vitro-derived (IP) plants at different times. Data are shown as the mean ± the standard error (n = 4). Asterix symbols (*) represent significant differences for pairwise comparisons according to one-way ANOVA.

In response to an over-accumulation of ROS, there is a number of enzymes that increase their activity to cope with oxidative stress. SOD is the first line of defense for oxidative stress, catalyzing the removal of O_2_^•−^ by dismutating it into O_2_ and H_2_O_2_; CAT converts the H_2_O_2_ into water and molecular oxygen (O_2_); whereas POX scavenges H_2_O_2_ in the extra-cellular space and vacuoles using phenolic as electron donors [49]. In this work, SOD activity increased over time in both plant types, especially in IPs, reaching a peak at day 56, which indicates a major preponderance of this enzyme at later stages. CAT activity levels slightly changed over the course of the experiment. However, the most noteworthy change corresponded to a nearly 3-fold increase in POX activity in NPs with respect to IPs (Figure 5), which can be considered as a mechanism triggered to ameliorate salinity-induced oxidative stress in these plants. Furthermore, the lower lipid peroxidation levels found in IPs would suggest a more intense antioxidant defense exerted by the specific activity of POX [55,56], thus minimizing oxidative stress associated with salinity.

Moreover, these enzymes not only safeguard cell components from damage, but also play a vital role in plant growth and development by participating in processes such as cell elongation, mitosis, senescence, detoxification of xenobiotics, conjugation of metabolites, and expression of stress-responsive genes, among others [57]. Therefore, a modulation of antioxidant enzymes may also be linked to variations in the growth and physiological responses of halophytic plants. In this context, a comprehensive characterization of the antioxidant system in halophytes remains limited. Additionally, species-specific salt-tolerance mechanisms have been identified, which vary according to the plant species and its native habitat [58,59].

## 4. Conclusions

This study represents the first evaluation of halophyte clones obtained through micropropagation under glasshouse conditions. Salt tolerance levels vary among halophytic species, with the specific habitat playing a crucial role in determining both the degree of salt tolerance and the adaptive strategies employed by different populations within the same species. While halophytes from diverse and heterogeneous habitats have been extensively studied, limited attention has been given to the unique characteristics of clonal halophytes [60].

In this sense, the differences found in the present study between IP and NP may rely on the existence of somaclonal variation in IP clones. IPs displayed superior biomass accumulation and desalting capacity. NPs showed more salt stress symptoms and a lower efficiency in photosynthesis, which correlated with higher levels of oxidative stress and with a remarkable induction of POX activity. It is, therefore, hypothesize that there is a higher incidence of salinity-related oxidative stress in the wild halophyte in comparison to the clones. This study supports the use of in vitro culture as a valuable approach for investigating the physiology of halophytes and emphasizes its potential in generating elite germplasm for desalination purposes.

## Figures and Tables

**Table 1:** Mineral nutrients in soil samples associated with Arthrocaulon macrostachyum plants. The symbols “*”, “**”, and “***” connote significant differences according to one-way ANOVA at 0.05, 0.01 and 0.001 levels, respectively. DW: dry weight. IP: in vitro-derived plants; NP: nursery plants.

	g Kg^−1^ DW				mg Kg^−1^ DW								
	Ca	Cl	Na	Fe	B	Cd	Co	Cr	Cu	Ni	Sr	Ti	Zn
IP	13.2	65.4	42.1	0.87	13.5	0.14	0.14	4.86	9.84	2.06	41.5	32.6	22.1
NP	16.4 *	70.0	38.3	1.40 **	19.1 *	0.18 *	1.30 **	9.25 *	30.2 **	3.84 *	71.2 ***	48.1 *	33.4 *

**Table 2:** Mineral nutrients in shoot samples of Arthrocaulon macrostachyum. The symbols “*”, “**”, and “***” connote significant differences according to one-way ANOVA at 0.05, 0.01, and 0.001 levels, respectively. DW: dry weight. IP: in vitro-derived plants; NP: nursery plants.

	g Kg^−1^ DW					mg Kg^−1^ DW					
	Cl	Na	NO_3_^−^	PO_4_^3−^	SO_4_^2−^	Li	Mn	Mo	Pb	Rb	Sr
IP	249	153	8.31	7.28	8.11	1.79	62.2	0.03	0.93	5.84	29.0
NP	195 **	150	19.2 **	15.3 *	13.1 *	2.96 *	47.1 *	1.65 *	0.50 *	9.65 *	50.4 ***

## Data Availability

Data generated during this study are available from the author upon request.
